# Efficient nitrogen remobilization and nitrogen partitioning to seeds in *Camelina sativa* grown under contrasting nitrogen regimes

**DOI:** 10.1186/s12870-025-07693-2

**Published:** 2025-12-29

**Authors:** Demian Dlakić, Patrick Munson, Chaofu Lu, Andreas Fischer

**Affiliations:** Department of Plant Sciences and Plant Pathology, Montana State University, Bozeman, MT United States of America

**Keywords:** Camelina, Nitrogen harvest index, Nitrogen use efficiency, Nitrogen remobilization efficiency, Senescence

## Abstract

**Background:**

Camelina (*Camelina sativa*) is an emerging intermediate oilseed crop, commonly grown between main crops for biofuel production. A related Brassica oilseed, canola, exhibits low nitrogen remobilization efficiency. To evaluate the possibility of a similar limitation in camelina, nitrogen remobilization and partitioning to seeds was examined by growing plants under starkly contrasting nitrogen regimes. Camelina variety ‘Suneson’ was grown on nutrient film tables, using nutrient solutions containing 6.5 mM (HN) or 0.65 mM (LN) nitrogen. Nitrogen was either removed at the onset of flowering or continued until plant maturity (four treatments). Single leaves, entire shoot and root systems, silicles (fruit pericarp), and seeds were harvested in weekly intervals starting with the onset of flowering, and analyzed for dry weight, carbon, and nitrogen contents.

**Results:**

Nitrogen treatments profoundly affected plant development, with reduced branching, reduced height, smaller leaves, and strongly accelerated shoot and leaf senescence observed under LN. Nitrogen was efficiently remobilized from leaves, shoots, and silicles to seeds, with residual nitrogen concentrations as low as 0.3% of dry weight observed in mature shoot systems of LN-removed plants, and a nitrogen harvest index of 0.6 to 0.7 depending on treatment. N regimes strongly influenced seed yield and composition, with seed N between 2.8% (HN-removed) and 4.5% of dry mass (HN-continued), while seed oil varied between 25% (HN-continued) and 45% (HN-removed).

**Conclusions:**

Camelina exhibits efficient nitrogen remobilization and partitioning to seeds under starkly contrasting nitrogen regimes, supporting its reputation as a low-input crop and justifying renewed interest in this emerging agricultural commodity.

**Supplementary Information:**

The online version contains supplementary material available at 10.1186/s12870-025-07693-2.

## Background

Camelina (*Camelina sativa*) is an underdeveloped oilseed crop in the Brassicaceae family that is being grown as an intermediate crop to produce biofuel, industrial bioproducts, and for human and animal consumption [1]. Camelina was most likely first cultivated in the Caucasus region [2], and is cold and drought tolerant, making it a candidate crop for the climate of the northern Great Plains, USA [3]. Camelina oil can provide a beneficial nutritional supplement for humans and animals due to high concentrations of omega-3 fatty acids and antioxidants [4, 5]. Camelina oil is also suited for use in biofuel applications, and many contemporary projects involving camelina oil are focused on potential use as an aviation biofuel. Tests with military and commercial airplanes have been successful, and compared to petroleum jet fuel, camelina-derived jet fuel blends can produce a 70% reduction in particulate emissions [6], a 50% reduction in carbon monoxide emissions [7], and 75% lower lifecycle carbon dioxide emissions [8]. All these results have increased interest in camelina as an intermediate crop for the northern Great Plains.

For most commercial field crops, nitrogen is the yield-limiting nutrient. The challenge of increasing yields while lowering nitrogen inputs has become a major focus of modern agriculture [9]. Camelina is less nitrogen intensive than many other field crops, but high yields still require significant fertilization in most field applications, making nitrogen fertilizers the most expensive input [10, 11]. In the Great Plains, camelina is usually grown in rotation, double cropping, or relay cropping with cereals as an alternative to fallow [12, 13]. However, the extra fertilizer costs discourage farmers from adding it to a cereal rotation, and are a major roadblock to increasing camelina acreage in the Great Plains [10.

In oilseed crops, nitrogen availability significantly influences both seed composition and yield [14]. Oil and storage proteins are the main components of oilseeds [15], with high protein typically associated with lower oil content [16]. Nitrogen availability also affects the rate of senescence in most field crops, particularly delaying senescence under conditions of high availability [17].

The effect of nitrogen on plant development and yield is often understood through the concept of nitrogen use efficiency (NUE). NUE describes how efficiently a plant uses nitrogen throughout its life cycle and contains several sub-components, including nitrogen remobilization efficiency (NRE). NRE quantifies the efficiency of nitrogen recycling between old (senescing) and young plant organs during vegetative growth, and specifically the efficiency of nitrogen remobilization to reproductive organs during flowering and seed filling [18]. Senescence and NRE are key topics for improving NUE in field crops [9, 19]. NRE is a known yield-limiting factor in canola [20, 21], with low NRE due to nitrogen loss in fallen leaves [22].

NRE has not previously been analyzed in camelina, and there are few studies investigating the plasticity of camelina growth, yield, and seed composition in response to nitrogen fertilization [23–25]. Therefore, the goal of this study was to investigate camelina NRE, and specifically to examine if camelina experiences low NRE as previously observed in canola [20, 21]. To this end, we analyzed NRE under strongly contrasting pre- and post-flowering nitrogen fertilization regimes, using a hydroponic culture system to strictly control nitrogen availability throughout the plant life cycle and particularly after the onset of flowering.

## Methods

### Plant material and growing conditions

Camelina variety ‘Suneson’ was chosen for the main trial of the study because it is a well-established cultivar developed for the northern Great Plains, and is also a model for molecular genetic studies in camelina [26]. Three additional lines (‘3’, ‘20’, and ‘53’) were selected for a second trial from a panel of 222 accessions grown in field studies based on visual variations in growth habit and other traits to expand the main Suneson trial [27]. Line ‘3’ is from Kyrgyzstan and is also known as ‘Kirgizskij’. Line ‘20’ is from Germany, and was obtained from the Deutsche Saatveredelung in Lippstadt, Germany; it is also known as ‘Lindo’. Line ‘53’ is also from Germany and was obtained from the Leibnitz Institute of Plant Genetics and Crop Plant Research in Gatersleben, Germany; it is also known as ‘PRFGL54’.

Camelina seeds in all trials were germinated in flats with 200 rockwool cubes manufactured by Grodan (Byhalia, MS) in a growth chamber set at 12 °C to encourage slow and even germination, a photosynthetically active radiation (PAR) of ~ 100 µmol m^− 2^ s^− 1^, and a 16 h/8 h day-night cycle. The flats of rockwool cubes were submerged in a half-strength formulation of the high nitrogen (HN) Hoagland solution (see below). After two weeks of germination, seedlings that had not yet developed true leaves were transferred to nutrient film tables (NFTs) in a climate-controlled growth room set at 22 °C and a humidity of ~ 60%. The growth room lights were Philips Greenpower toplighting 200-400v LED rails providing between 300 and 500 µmol m^− 2^ s^− 1^ PAR at the level of growing plants, with 16 h/8 h day-night cycles maintained. The hydroponic system used was an eight channel NFT system. Each channel was 2.5 cm deep and 10.2 cm wide, with 2.5 cm square holes in the covers for each plant. Each channel held 18 plants and a total of 144 plants were grown under each nutrient treatment (four treatments for the main Suneson trial and two treatments in the expanded trial). The nutrient solution reservoir was a black 100 L covered tub. The nutrient solution was cycled through the channels using a 1400-liter per hour pump (Ecoplus: Vancouver WA), and the reservoir was aerated by two air stones fed by a Tetra whisper aquarium air pump (model 60).

### Nutrient solution

The nutrient solution used in each trial was a modified Hoagland solution that was completely changed weekly throughout the duration of each experiment; the composition is provided in Supplemental Table S1. The treatments were chosen to advance the physiological understanding of nitrogen remobilization efficiency in camelina, and to determine how nitrogen availability at pre- and post-flowering stages influences yield components. The high nitrogen (HN) treatment represents a full strength Hoagland recipe, containing 6.5 mM nitrogen in a combination of 5.5 mM nitrate and 1 mM ammonium [28]. The low nitrogen (LN) treatment used the same recipe, but all nitrogen minerals were lowered by a factor of 10. The concentration of nitrogen was reduced to 0.65 mM in a combination of 0.55 mM nitrate and 0.10 mM ammonium. Potassium nitrate (KNO_3_) and calcium nitrate (Ca[NO_3_]_2_) were replaced with KCl and CaCl_2_ to maintain identical K and Ca levels. Levels of additional Cl^−^ in the solution were 4.05 mM under LN and 4.5 mM in N-removed treatments, below the threshold of potential chloride toxicity [29, 30]. Both the HN and LN treatments were either continued after the onset of flowering, or the nitrogen in the solution was removed after flowering, using the N-removed recipe shown in Table S1. This created four treatments: HN-removed, HN-continued, LN-removed, and LN-continued.

### Tissue collection

Plants were harvested weekly starting with the onset of flowering (recorded as ‘week zero’). The beginning of flowering was defined as the point when half the plants on the NFT had at least one flower that had completely opened and revealed petals. In all 4 trials, this stage occurred on either the 42^nd^ or 43^rd^ day after the seeds were germinated and after four complete nutrient solution changes. Plants (20 plants per sampling time point) were harvested following a randomized complete block design with each channel of the NFT serving as a block. The first 10 plants had their entire root systems removed, rinsed to remove any residual nutrient solution, and placed in a paper bag for drying; all aboveground tissue was then photographed. After photographing, all silicles were removed (if present, regardless of developmental stage) and placed in envelopes. The remaining aboveground tissue (stems and leaves, including fallen leaves) was defined as the ‘shoot system’ and was placed in a paper bag for drying. For the second series of 10 plants, each leaf of the primary stem was removed and photographed in a line. Leaves 2, 8, 13, and 19 were placed into 15 ml test tubes for drying. Plants were harvested until maturity, defined as the point when all main and secondary stems had visually senesced. When growing lines 3, 20, and 53, Suneson was included for comparison. These plants were only harvested when all lines had reached maturity (week fourteen after flowering under HN-continued, and week five for HN-removed); tissues were collected in the same manner as the Suneson-only trials.

### Tissue analysis

The root and shoot systems in paper bags were dried at 43 °C and less than 10% humidity for at least a week. Silicles were dried at room temperature for at least two weeks and then crushed and filtered through 1 mm and 0.8 mm sieves to separate all present seeds. The dry tissues were then weighed and ground. Root system, shoot system, and silicle tissues were ground in a Thomas Wiley Mill Model 4 (Swedesboro, NJ) and filtered through a 0.5 mm sieve. Seeds were stored in 15 ml test tubes and weighed before analysis. Individual leaves (2, 8, 13, and 19) were ground by hand using a Foredom model DD rotary hand drill with plastic pestles in a 1.5 ml Eppendorf tube. Analysis of total carbon and total nitrogen was performed by a Costech Analytical Technologies ECS 4010 elemental combustion analyzer at the Environmental Analytical Laboratory at Montana State University, Bozeman, MT and at the Stable Isotope Core Laboratory at Washington State University, Pullman, WA. Samples consisting of ~ 2 mg dry tissue (or 2–3 seeds) were used for each elemental analysis.

Seed oil concentration was determined by NMR analysis (Oxford Instruments model MQC, Abingdon, UK) of ~ 2 mg samples at 40 °C based on a standard curve of reference camelina oil. Fatty acid analysis was conducted via gas chromatography (Shimadzu model GC- 2010, Kyoto, JP) of fatty acid methyl esters as described by Lu et al. [31]. Statistical analyses were completed in R; 95% confidence intervals were two-sided, and all compact letter displays were produced via one-way ANOVA and Tukey’s honest significance test at a 95% confidence interval using the multcomp R package [32]. Graphs were made in R with the ggplot2 package [33].

## Results

### Nitrogen regimes determine plant architecture and leaf senescence rate

**Fig. 1:**
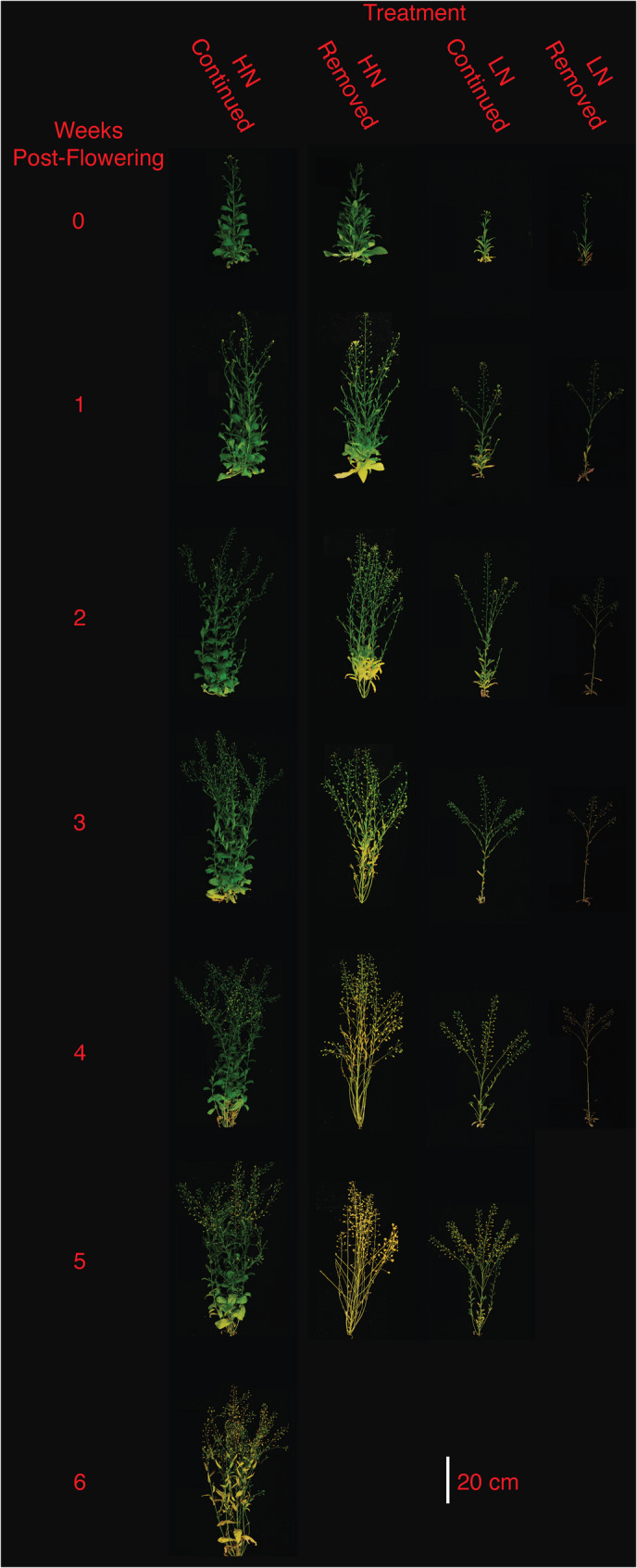
Camelina plants grown under four nitrogen regimes are shown in weekly intervals, starting with the onset of flowering (week zero). One representative harvested plant is shown in each case

**Fig. 2:**
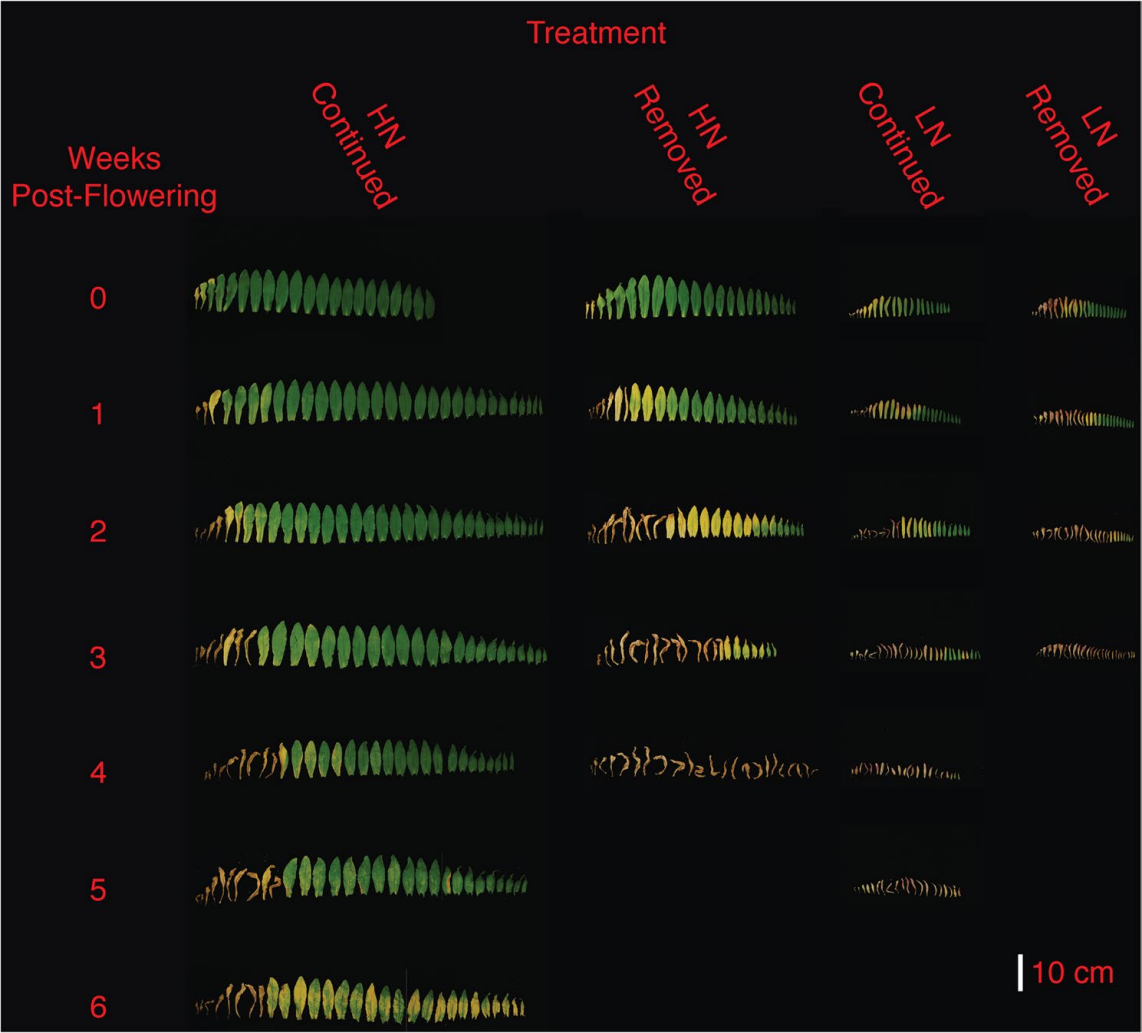
Main stem camelina leaves from plants grown under four nitrogen regimes are shown in weekly intervals, starting with the onset of flowering (week zero). Leaves from one representative harvested plant are shown in each case

Across all treatments, plants began flowering on the 42^nd^ or 43^rd^ day after germination. Plants reached maturity at 70 days (LN-removed), 78 days (HN-removed and LN-continued), and 140 days (HN-continued). When examining entire shoot systems (Fig. 1), it was visually apparent that nitrogen treatments profoundly affected overall plant morphology. LN resulted in visibly reduced branching, plant height, and leaf size, and strongly accelerated shoot and leaf senescence (Figs. 1 and 2). In both N-removed and N-continued treatments, a larger fraction of the shoot system and leaves had senesced under LN than HN at week zero (Figs. 1 and 2). Additionally, under both LN and HN, observable shoot and leaf senescence was greatly accelerated by nitrogen removal at the onset of flowering. At week zero the LN-continued treatment displayed a higher number of visibly senesced leaves than the HN-removed treatment, but by week four the HN-removed plants had completely senesced while LN-continued plants still showed some green shoots and leaves (Figs. 1 and 2). The largest visual difference in leaf senescence rate was seen between the HN-continued and LN-removed treatments (Fig. 2).

### Camelina leaves, shoots, and silicles efficiently remobilize nitrogen

**Fig. 3:**
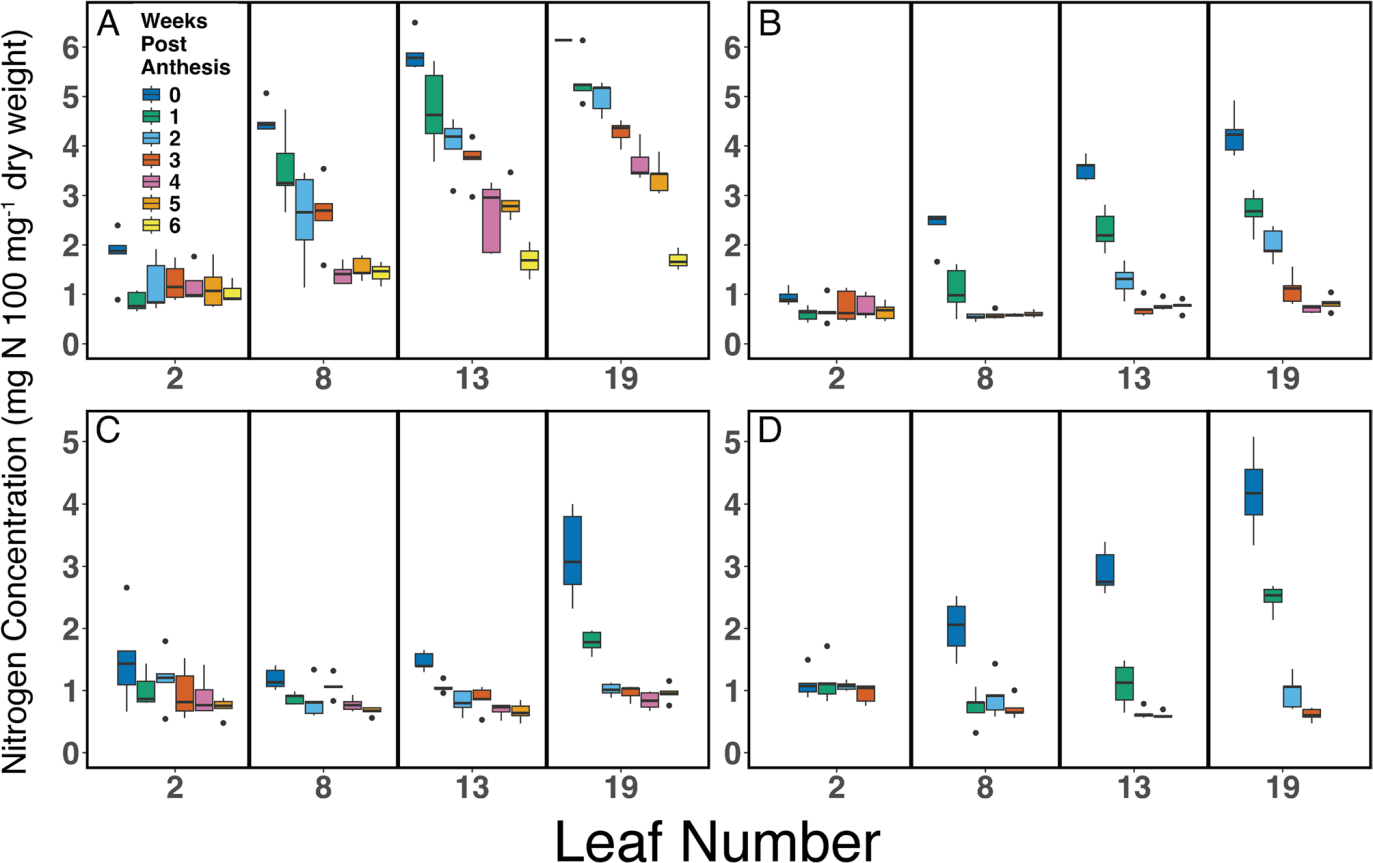
Nitrogen concentrations in leaves 2, 8, 13, and 19 of camelina main stems are shown in weekly intervals, starting with the onset of flowering (week zero). Lower numbers designate older leaves, with leaf 1 as the first true leaf. **A**, HN-continued; **B**, HN-removed; **C**, LN-continued; **D**, LN-removed. Boxplots (*n* = 5) display median and first and third quartiles, whiskers extend to 1.5 times the interquartile range, and outliers are displayed as dots

When examining nitrogen concentrations in main stem leaves (Fig. 3), the patterns of nitrogen removal matched visual leaf senescence symptoms (Fig. 2). At week zero, nitrogen percentage in leaf 2 was at or below 2% across all treatments. It quickly decreased to 1% or lower in subsequent weeks (Fig. 3). Higher (younger) main stem leaves displayed larger changes in nitrogen concentration as leaf senescence initiated after the onset of flowering. Across all treatments, nitrogen percentages in leaves 8, 13, and 19 decreased, but the rate varied between treatments (Fig. 3). HN-continued produced leaves with the lowest senescence rate; leaves 8, 13, and 19 still displayed nitrogen percentages above 1% by six weeks after the onset of flowering (Fig. 3 A). In all treatments except HN-continued, nitrogen percentages in all leaves were below 1% at five weeks post-flowering, regardless of the percentage at week zero.

**Fig. 4:**
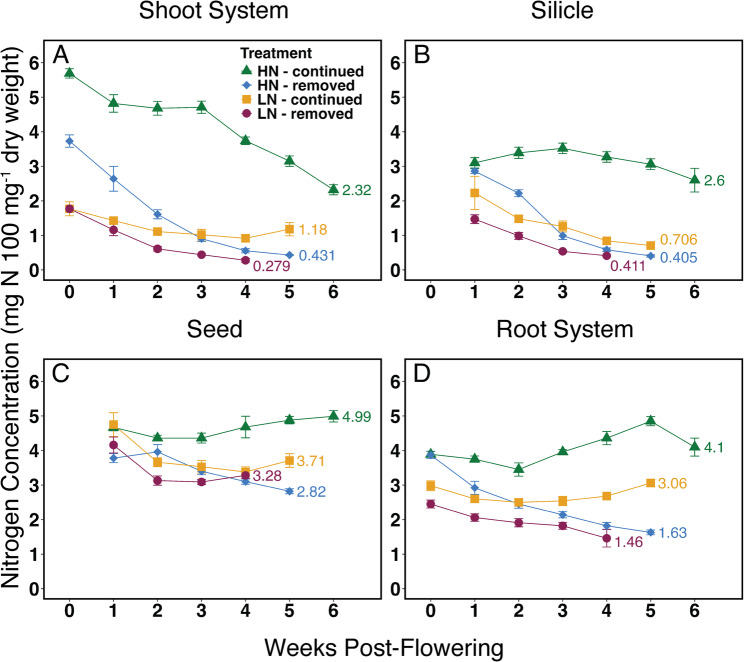
Nitrogen concentrations in camelina plant organs are shown in weekly intervals, starting with the onset of flowering (week 0). **A**, shoot systems including all stems and leaves; **B**, silicles (fruit pericarp); **C**, seeds; **D**, root systems. Mean values (*n* = 10) and error bars representing a 95% confidence interval are shown

The shoot system (including all stems and leaves) followed a pattern of nitrogen remobilization which was similar to individual leaves. After the onset of flowering, nitrogen concentrations in both N-removed treatments dropped rapidly for the first two weeks, and continued to decrease more slowly over the following two to three weeks (Fig. 4 A). Shoot nitrogen percentages reached < 0.5% at weeks five (HN-removed) and four (LN-removed) post-flowering (Fig. 4 A). HN-continued displayed a drop in nitrogen percentage one week after flowering, which stabilized between weeks one and three (Fig. 4 A), before continuing to decline to a value of 0.87% at week fourteen (Table S2). The LN-continued treatment exhibited a declining nitrogen percentage until four weeks post-flowering followed by an increase, which was the only increase in shoot system nitrogen percentage observed across all treatments, and led to the only final shoot nitrogen concentration > 1% (Fig. 4 A)

Nitrogen concentrations in silicles showed patterns which are similar to the other aboveground tissues. The N-removed treatments showed substantial remobilization of silicle (fruit pericarp) nitrogen, and both treatments produced a nitrogen percentage below 1% at week three post-flowering (Fig. 4B). Nitrogen percentages continued to decrease to ~ 0.4% at weeks four and five under the LN-removed and HN-removed treatments, respectively (Fig. 4B). The LN-continued treatment followed a similar trend; nitrogen percentage in silicles decreased to ~ 0.7% at five weeks post-flowering (Fig. 4B). The HN-continued plants showed slightly increasing silicle nitrogen percentages during the first weeks before nitrogen concentrations began to fall (Fig. 4B). At fourteen weeks post-flowering, the HN-continued silicles contained ~ 1.2% nitrogen, higher than in shoots (Table S2). All treatments except HN-continued produced silicles with < 1% nitrogen at the end of senescence, similar to individual leaves. 

Seeds began to form in the first week after the onset of flowering, and at week one post-flowering, all seeds contained between ~ 3.5% and ~ 5% nitrogen which remained largely unchanged as more seeds were produced and seed filling progressed (Fig. 4 C and Table S2). The lowest nitrogen concentration was seen in mature seeds of plants grown under HN-removed (~ 2.8%) (Fig. 4 C). Nitrogen percentages decreased in root systems under the N-removed treatments, and increased after two weeks post-flowering in HN-continued (Fig. 4D). Root nitrogen percentages in mature plants were higher than aboveground vegetative tissues across all treatments (Fig. 4D).

### Nitrogen remobilization is efficient in multiple camelina lines

**Fig. 5:**
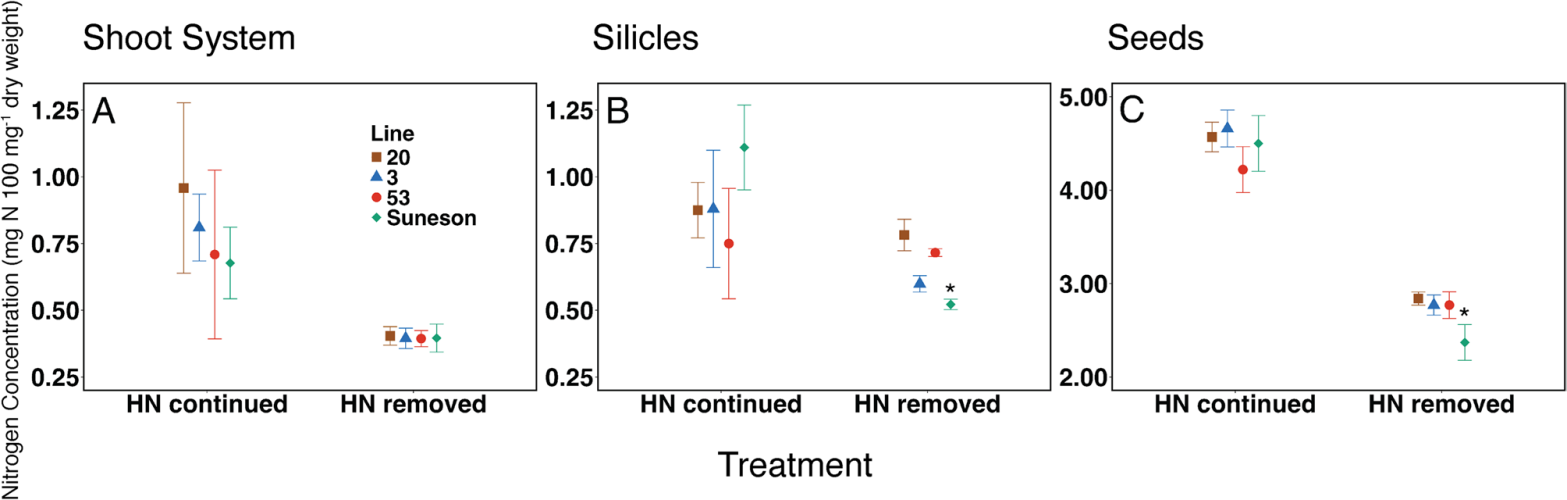
Residual nitrogen concentrations in camelina plant organs of three representative lines and Suneson are shown at maturity (fourteen weeks after the onset of flowering under HN-continued, and five weeks under HN-removed). **A**, shoot systems; **B**, silicles; **C**, seeds. Mean values (*n* = 5) and error bars representing a 95% confidence interval are shown. Stars indicate significant differences (*p* < 0.05; determined by Tukey’s honest significance test) between lines under the same nitrogen treatment

Analysis of nitrogen allocation was expanded to include three additional lines (see ‘Materials and Methods’). Residual nitrogen concentrations in shoot systems of mature plants were similar in all germplasm tested (Fig. 5 A). Nitrogen concentrations were also similar in silicles, although Suneson exhibited the lowest silicle N concentration under HN-removed (Fig. 5B). Seed N concentrations were similar across lines under HN-continued, and Suneson was the lowest under HN-removed (Fig. 5 C). Together, data presented in Fig. 5 suggests that nitrogen remobilization and allocation patterns analyzed in detail for variety Suneson (Figs. 3 and 4) are representative of those in other camelina lines.

### Whole plant nitrogen partitioning controls seed yield and seed composition

**Fig. 6:**
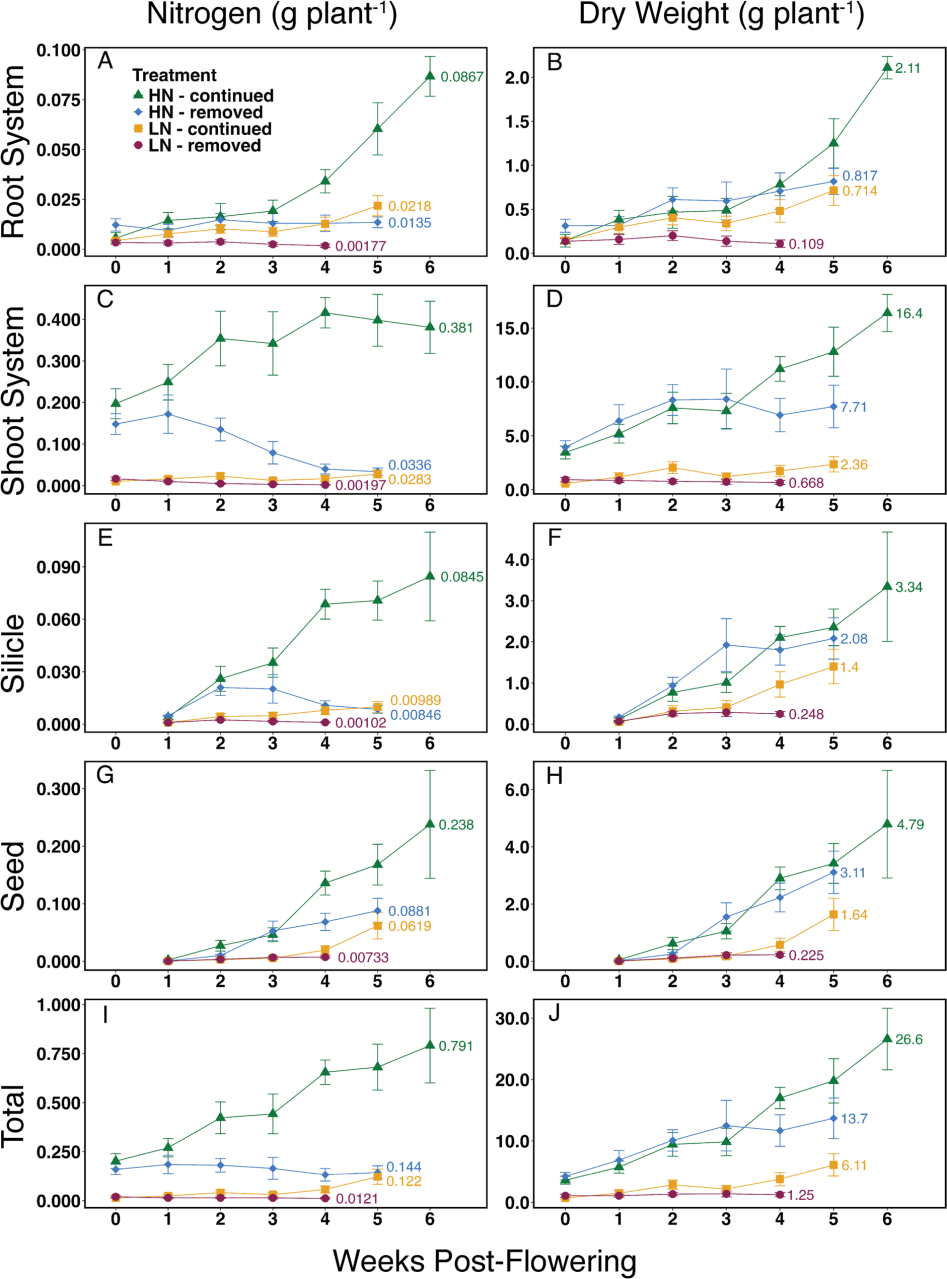
Nitrogen mass and dry weight (biomass) of camelina plant organs are shown in weekly intervals, starting with the onset of flowering (week 0). Left panels show nitrogen mass, and right panels show dry weight. **A**, **B**, root systems; **C**, **D**, shoot systems including all stems and leaves; **E**, **F**, silicles (fruit pericarp); **G**, **H**, seeds; **I**, **J**, total plant nitrogen mass and dry weight. Mean values (*n* = 10) and error bars representing a 95% confidence interval are shown

Tracking changes in tissue nitrogen concentrations provides an understanding of camelina NRE, while analysis of dry mass and nitrogen mass in the different plant parts over time delineates nitrogen partitioning and yield formation [34]. N-continued treatments showed increasing root system nitrogen mass post-flowering, while N-removed treatments exhibited little change (Fig. 6 A, Fig. S2A, Fig. S3A, and Fig. S4A). Root system carbon mass and biomass increased over time across all treatments except LN-removed, where it began to decrease after week two (Fig. 6B, Fig. S1A, Fig. S2B, Fig. S3B, Fig. S4B). Root system biomass was below 10% of total plant biomass in all treatments except LN-continued (Fig. 6B and J and Fig. S2B).

Shoot nitrogen mass partitioning and biomass were sharply different between LN and HN treatments, as HN plants had much larger shoot systems and higher shoot/root ratios than LN plants (Figs. 1 and 6). After the first week post-flowering, shoot nitrogen mass decreased in the HN-removed plants until it reached the level of the LN-continued treatment, below 40 mg plant^− 1^ (Fig. 6 C, Fig. S2C, Fig. S3C). The HN-continued plants exhibited an increase of shoot nitrogen mass post-flowering that plateaued after week four (Fig. 6 C). In the LN treatments, shoot nitrogen mass deviated after week three, when the LN-continued plants increased shoot nitrogen mass to above 20 mg plant^− 1^ (Fig. 6 C, Fig. S2C, Fig. S4C). Shoot biomass at maturity was over half of the total plant biomass in all treatments except LN-continued (Fig. 6D and J, and Table S2). There was a distinct separation in shoot biomass between HN and LN treatments, with HN-continued plants reaching > 25 g plant^− 1^ (Table S2) and HN-removed reaching > 7 g plant^− 1^, while both LN treatments remained below 3 g plant^− 1^ (Fig. 6D). Shoot carbon mass followed a similar pattern to dry mass across all treatments, and was approximately one third of the total plant biomass (Fig. 6D and Fig. S1B).

**Fig. 7:**
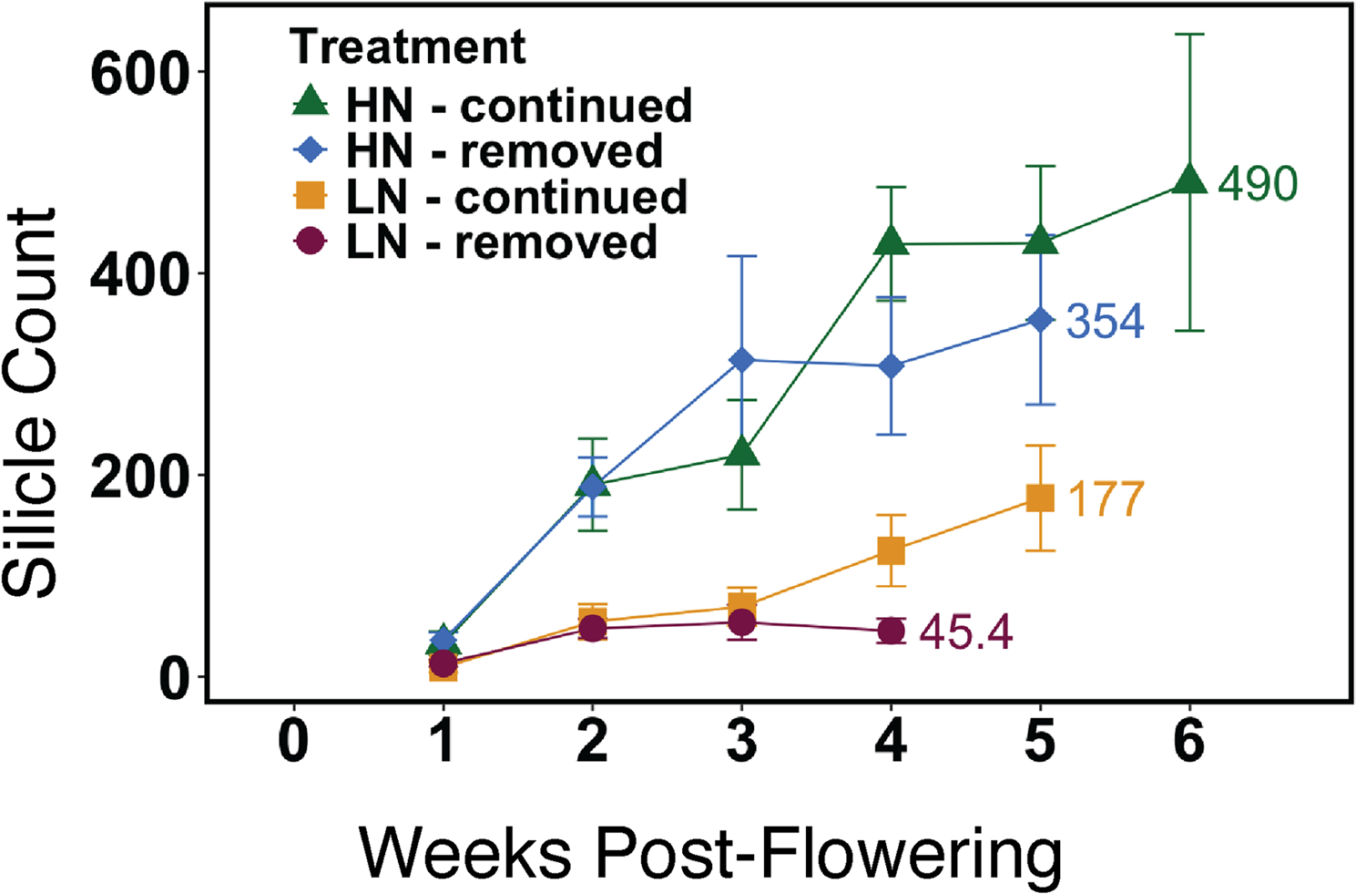
Silicles per plant, shown in weekly intervals starting at week 1 after the onset of flowering. Mean values (*n* = 10) and error bars representing a 95% confidence interval are shown

Nitrogen mass in silicles increased as silicles formed and nitrogen was remobilized from shoot tissues after week 0, but remained lower than nitrogen in the other plant parts (Fig. 6). Silicle nitrogen mass continued to rise until the end of senescence in both N-continued treatments (Fig. 6E and S2E), and began to decrease after week two in the N-removed treatments (Fig. S3E, and S4E). Silicle mass and number increased until senescence arrested plant growth (Figs. 6 F and 7; Table 1)

**Table 1:** Mean values (*n* = 10) of seed oil and yield traits at maturity. HN-continued is shown at fourteen weeks, HN-removed and LN-continued are shown at five weeks, and LN-removed is shown at four weeks after the onset of flowering. Different letters indicate significant differences between treatments (*p* < 0.05) based on Tukey’s honest significance test. TSW, thousand seed weight

	Seed Yield (g plant^− 1^)	TSW (g)	Silicles (per plant)	Seed Oil (%)	Oil Yield (g plant^− 1^)	Harvest Index	Nitrogen Harvest Index
**HN-continued**	9.59^C^	1.23^C^	874^C^	25.44^A^	2.45^C^	0.23^B^	0.59^A^
**HN-removed**	3.11^B^	0.91^B^	354^B^	44.83^C^	1.39^B^	0.24^B^	0.68^B^
**LN-continued**	1.64^AB^	0.99^B^	177^A^	38.11^B^	0.64^A^	0.30^C^	0.61^A^
**LN-removed**	0.23^A^	0.73^A^	45^A^	37.26^B^	0.08^A^	0.20^A^	0.72^B^

### Nitrogen regimes influence yield indices, seed composition, and oil quality

Patterns of seed nitrogen allocation over time diverged from those of all other plant organs. Once seeds began to form after week one post-flowering, seed nitrogen mass increased strongly until plant maturity in all treatments (Fig. 6G; Fig. S2G, S3G, and S4G). Unlike seed nitrogen mass, seed carbon mass and dry weight (seed yield) were < 50% of total plant carbon and biomass, but followed a similar pattern of increasing steadily from week one until plant maturity (Fig. 6 and Fig. S1). Continued nitrogen availability after the onset of flowering resulted in higher total seed yields under both HN and LN conditions. The difference in seed yield between LN-removed and HN-removed was ~ 14-fold, while an ~ 6-fold difference was observed between the LN-continued and HN-continued treatments (Table 1).

HN-continued produced the highest average oil yield, significantly higher than HN-removed (Table 1). Among the LN treatments, LN-continued produced a higher average oil yield than LN-removed (Table 1). However, this pattern of similar seed yield and seed oil yield obscured differences in seed composition. Plants grown under the HN-removed treatment produced seeds with a considerably higher oil concentration (44.8%) than plants grown under HN-continued (25.4%) (Table 1). In contrast, seeds from plants grown under LN contained similar oil concentrations of 37.3% (LN-removed) and 38.1% (LN-continued) (Table 1). This trend in seed oil was reflected in seed carbon concentrations (Fig. S5C). 

Harvest index (HI) is a descriptive statistic that reflects resource partitioning to seeds by measuring the fraction of aboveground biomass that is contained in seeds [35]. The HN treatments showed no significant difference in HI when nitrogen was removed or continued (Table 1). The highest HI was seen for the LN-continued treatment, significantly higher than the LN-removed treatment (Table 1). HI can also be measured in terms of nitrogen, as nitrogen harvest index (NHI). NHI compares the ratio of nitrogen present in seeds to nitrogen present in all aboveground biomass, and a high NHI indicates high nitrogen remobilization efficiency [36]. All treatments produced an NHI of >0.59 (Table 1). The HN treatments differed significantly in NHI; nitrogen removal or continuation led to an NHI of 0.68 and 0.59, respectively (Table 1). The LN-removed treatment produced a similar value to HN-removed (0.72), and LN-continued presented a significantly lower NHI (0.61), similar to HN-continued (Table 1).

Oil composition was also significantly affected by nitrogen treatment. In the HN-continued treatment, saturated fatty acids including palmitic acid (16:0), stearic acid (18:0), and arachidic acid (20:0) contributed a larger fraction than is seen under HN-removed (Table 2). In contrast, the monounsaturated oleic acid (18:1) was more abundant in seeds harvested from plants grown under HN-removed than under HN-continued conditions (Table 2). All other fatty acids that significantly contribute to camelina oil composition including linoleic acid (18:2), linolenic acid (18:3), and eicosenoic acid (20:1) were present at similar percentages between the HN-continued and HN-removed treatments. Oil composition differences between the LN-removed and LN-continued treatments were minor, with only stearic acid (18:0) and linoleic acid (18:2) displaying a significant difference in percentage (Table 2). Thus, the two LN treatments produced seeds with a similar composition despite a large difference in total seed yield per plant and thousand seed weight (Fig. 6H; Tables 1 and 2, Fig. S2H and S4H). In contrast, seed lipid concentration, lipid composition, and nitrogen concentration were substantially different between the two HN treatments.

**Table 2:** Mean values (*n* = 10) of seed oil analysis. Values represent fatty acid types as a percentage of the whole extraction. HN-continued is shown at fourteen weeks, HN-removed and LN-continued are shown at five weeks, and LN-removed is shown at four weeks after the onset of flowering. Different letters indicate significant differences between treatments (*p* < 0.05) based on Tukey’s honest significance test

Fatty Acid	16:0	16:1	18:0	18:1	18:2	18:3	20:0	20:1	20:2	20:3	22:0	22:1
**HN-continued**	6.84^B^	1.78^B^	2.76^C^	12.04^A^	17.52^A^	39.49^A^	1.70^B^	12.12^A^	1.71^A^	1.41^A^	0.44^B^	2.19^B^
**HN-removed**	5.58^A^	1.03^A^	1.60^AB^	15.80^C^	17.34^A^	39.87^A^	0.49^A^	12.14^AB^	2.55^B^	1.63^B^	0.25^A^	1.71^A^
**LN-continued**	6.22^B^	1.15^A^	1.83^B^	13.35^B^	17.82^A^	39.64^A^	0.60^A^	12.81^BC^	2.62^B^	1.61^B^	0.30^A^	2.05^B^
**LN- removed**	6.34^B^	1.03^A^	1.59^A^	13.77^B^	19.73^B^	37.45^A^	0.61^A^	12.88^C^	2.71^B^	1.57^B^	0.31^A^	2.01^B^

In summary, the HN-removed treatment produced seeds with the highest oil and carbon concentrations alongside the lowest nitrogen concentration and an average seed weight of 0.91 mg. Conversely, the HN-continued treatment produced seeds with the lowest oil and carbon concentrations alongside the highest nitrogen percentage and an average seed weight of 1.2 mg. The LN plants produced similar oil, carbon, and nitrogen concentrations that fall between the two HN treatments. The only significantly different seed trait under LN conditions was seed weight; plants grown under LN-removed conditions produced the smallest seeds of all treatments (0.73 mg) (Table 1). Regardless of nitrogen treatment, all seeds contained > 2.8% nitrogen (Fig. 4 C; Table S2). Plants that were nitrogen stressed before or during flowering also produced seeds with a higher oil percentage, significantly higher than the low (25.4%) oil concentration found under HN-continued (Table 1). The low oil concentration under HN-continued was offset by increased seed yield that led to the highest oil yield per plant of all treatments.

## Discussion

### Nitrogen remobilization efficiency is higher in camelina than in canola and arabidopsis

Efficient nitrogen remobilization from senescing plant organs to vegetative (e.g., developing leaves) and reproductive sinks (seeds) enhances plant NUE. Conceptually, nitrogen in plants can be divided into mobilizable and non-mobilizable fractions, with the latter consisting of cell wall proteins, components of the remobilization machinery (e.g., proteases and transporters), and organelles maintaining cell function and energy metabolism (nuclei, mitochondria) [37]. Realized nitrogen remobilization is influenced by the environment and plant development, and thus may be substantially lower than the theoretical limit dictated by the strictly non-mobilizable fraction.

The present study assessed the range of camelina NRE under contrasting nitrogen conditions using a hydroponic system to strictly control nitrogen availability both before and after the onset of flowering. Remobilization from aboveground tissues and NHI were used to assess NRE. Main stem leaves 2, 8, 13, and 19 all terminated at residual nitrogen levels below 1%; only leaves from the HN-continued treatment maintained levels above 1% by six weeks after the onset of flowering, but shoot systems of these plants reached ~ 0.9% when plants were fully mature at fourteen weeks (Fig. 3, Table S2). These values indicate highly efficient nitrogen remobilization from leaf tissues, defined as residual nitrogen < 1% [37]. NRE from leaves and shoots of LN-removed and HN-removed plants is very high, with values of 0.6%−0.95% in leaves and < 0.5% in shoots (Fig. 4 A and Table S3) meeting the ~ 0.7% residual nitrogen threshold used to define ‘complete’ remobilization (i.e., with only strictly non-mobilizable nitrogen left) [37]. In canola, NRE is considered low, with significant residual nitrogen in dropped leaves [22, 38–40] and pods [40, 41] (Table S3). It is consistently at or above 1% [39, 40] and reaches values well above 2% of dry mass [22, 40, 42] under high nitrogen fertilization in field and hydroponic studies (Table S3). The comparison of residual leaf N concentrations reported for canola [22, 39, 40, 42] and *Arabidopsis thaliana* (Arabidopsis) [43] with values for camelina reported here suggests that camelina remobilizes leaf nitrogen more efficiently, especially when nitrogen is not limiting (Table S3).

The camelina shoot system, representing the largest fraction of plant biomass during the vegetative stage, also displays efficient nitrogen remobilization. In all treatments except LN-continued the residual nitrogen percentage is 0.9% or lower, indicating NRE similar to or higher than individual leaves (Fig. 4 and Table S2). Low residual nitrogen concentrations were also found in the shoot systems lines 3, 20, and 53 under HN-removed and HN-continued conditions (Fig. 5 A). These values are comparable to residual nitrogen in field and greenhouse grown canola [39, 41] and greenhouse grown Arabidopsis [43, 44] shoot systems under low nitrogen availability, but lower than both species under a high nitrogen treatment (Table S3).

Silicles also contain residual nitrogen concentrations below 1.2% in all treatments, and the smallest nitrogen fraction of all organ groups (Figs. 4 C and 6 and Table S2). In lines 3, 20, and 53, residual nitrogen concentration in silicles was more variable than in shoots, but still below 1.1% (Fig. 5B). Compared to similar studies in canola, residual nitrogen in camelina silicles is lower under high nitrogen treatments, and similar under low nitrogen [40, 41] (Table S3). Silicles act as an important source of assimilated carbon during seed filling, especially after stem and leaf tissues have senesced, as shown in a previous study [45]. The different nitrogen treatments and varied rates of senescence led to significant differences in the number of silicles, rate of silicle development, duration of silicle development, and ultimately seed yield. The link between seed formation and pod development in Brassicaceae is well documented [46]. Camelina appears to follow this pattern of push-pull dynamics between leaves and silicles with high efficiency as remobilization occurs. Roots are the only organ group that does not display high NRE post-flowering, which is consistent with previous studies of camelina [47]. Nitrogen in root systems did not decrease except for the LN-removed treatment, suggesting that roots are not a significant source of nitrogen remobilization during seed filling (Figs. 4D and 6 A, Fig. S4A).

Seeds are the primary nitrogen sinks during reproductive growth. Seed nitrogen concentrations at maturity varied between 2.8% (HN-removed) and 4.5% (HN-continued), demonstrating that, despite high NRE, continued N availability during reproductive growth strongly influences this trait (Fig. 4 C, Table S2). Higher seed protein content often positively influences seed vigor [48], suggesting a negative impact of post-flowering nitrogen limitation on seed quality.

NHI is a useful metric to assess NRE during reproductive growth. Camelina NHI was relatively high across all treatments when compared to other dicot field crops [36]. Canola has a low NHI that varies with nitrogen treatment, representing another indicator of low NRE. NHI values in camelina (0.59–0.68 under HN and 0.61–0.72 under LN) were substantially higher than those reported in greenhouse grown canola under high nitrogen treatments and/or in N-inefficient cultivars (consistently below 0.4), and were similar or higher than N-efficient cultivars grown under low nitrogen treatment, which can reach between 0.5 and 0.6 [49, 50] (Table S3). Camelina NHI values were also higher than those reported for Arabidopsis [44, 51] (Table S3). Both N-removed treatments produced a significantly higher NHI than the continued treatments, and NHI was lowest under HN-continued. This suggests that nitrogen availability during reproductive growth has a larger effect on NHI (and NRE by extension) than nitrogen availability during vegetative growth

### Nitrogen regimes determine seed yield

Camelina has been promoted as a low-input crop [52], but, unsurprisingly, yield is increased in response to high nitrogen conditions [25, 53]. Plants reached maturity within the normal range for dryland conditions (60–100 days) [3, 11, 54], except under HN-continued (140 days), which resulted in delayed maturity due to the continuous supply of water and high nitrogen post-flowering. In the present study, seed yield per plant is strongly improved in response to greater nitrogen availability (Table 1), likely due to enhanced growth/branching, delayed senescence, and an extended reproductive growth phase due to the continuous supply of water and high nitrogen post-flowering (Figs. 1 and 2). Seed yield closely followed silicle number (Fig. 7) across all treatments. The large differences between N-removed and N-continued conditions under both LN and HN suggest that, despite efficient N remobilization, continued N availability from root uptake post-flowering substantially contributes to camelina reproductive growth and seed yield

###  Nitrogen regimes influence seed composition and oil yield

While seed yield followed expected trends related to nitrogen regimes, seed composition also responded to nitrogen availability. LN-grown plants produced similar oil concentrations, and were within the typical range based on reports from field studies (36%−40%) [23, 55]. Oil concentration under HN-removed was higher (44.8%) than the reported range, while HN-continued produced a concentration substantially below that range (25.4%), suggesting that oil concentration in camelina grown under high fertilization is strongly influenced by nitrogen availability after flowering. Increased nitrogen fertilization leading to seeds with a higher protein but lower oil concentration has previously been seen in field studies of camelina [24, 53] and other oilseed crops including canola [56, 57]. The HN-removed plants senesced faster, yielded considerably less, but produced seeds with a much higher seed oil concentration (Figs. 1 and 2; Table 1). The HN-removed treatment also produced oils with a lower fraction of saturated fatty acids than the HN-continued treatment. This change of oil composition under high nitrogen has also seen in canola [58]. Therefore, nitrogen availability during reproductive growth had a significant effect on seed oil concentration, yield, and composition.

## Conclusions

Despite being less nitrogen intensive than some other field crops, it is apparent that camelina suffers a yield penalty when grown in fields with low nitrogen availability [23]. Currently, fertilizer cost represents the primary obstacle to the profitability of camelina grown on the northern Great Plains [10], limiting acreage. In the present study, camelina exhibited a high NRE compared to that reported in canola, justifying renewed interest in camelina as a low-input intermediate oilseed crop. Data presented in this study also enhance our understanding of the relationships between nitrogen availability, seed yield, and seed composition. Further research is needed to decipher the molecular mechanisms governing NUE and to provide suggestions for camelina plant management and sustainable production.

## Supplementary Information

Supplementary Material 1. Supplementary data contains three tables; the first table lists the composition of the nutrient solutions, the second table provides nitrogen concentrations and dry weights for the HN-continued treatment 14 weeks after the onset of flowering, and the third table compares residual nitrogen values in camelina shoots, leaves, and silicles with literature data for canola and Arabidopsis. The first supplementary figure (S1) shows the carbon masses in plant organs for all four treatments at weekly intervals. Supplementary figures S2-S4 contain the nitrogen masses and dry weights for HN-removed, LN-continued, and LN-removed individually for additional clarity. Supplementary figure S5 contains the carbon concentration in plant organs for all four treatments at weekly intervals.

## Data Availability

The data generated in this study is available from the corresponding author upon reasonable request.

## Abbreviations

HN: High nitrogen (6.5 mM nitrogen)
LN: Low nitrogen (0.65 mM nitrogen)
NUE: Nitrogen use efficiency
NRE: Nitrogen remobilization efficiency
NFT: Nutrient film table
HI: Harvest index
NHI: Nitrogen harvest index
PAR: Photosynthetically active radiation
TSW: Thousand seed weight
