# Supplementary material for: Efficient nitrogen remobilization and nitrogen partitioning to seeds in Camelina sativa grown under contrasting nitrogen regimes
Source: BMC Plant Biol. 2025 Dec 29;25:1740. doi: 10.1186/s12870-025-07693-2 (PMC12752063; doi:10.1186/s12870-025-07693-2)
Supplement: Supplementary file 1 — Supplementary Material 1. Supplementary data contains three tables; the first table lists the composition of the nutrient solutions, the second table provides nitrogen concentrations and dry weights for the HN-continued treatment 14 weeks after the onset of flowering, and the third table compares residual nitrogen values in camelina shoots, leaves, and silicles with literature data for canola and Arabidopsis. The first supplementary figure (S1) shows the carbon masses in plant organs for all four treatments at weekly intervals. Supplementary figures S2-S4 contain the nitrogen masses and dry weights for HN-removed, LN-continued, and LN-removed individually for additional clarity. Supplementary figure S5 contains the carbon concentration in plant organs for all four treatments at weekly intervals. [file 12870_2025_7693_MOESM1_ESM.docx]

**Supplementary Tables and Figures**

**Table S1**

Recipes for high nitrogen (HN) (6.5 mM nitrogen), low nitrogen (LN) (0.65 mM nitrogen), and nitrogen-removed nutrient solutions used in this study.

| Solution | Chemical | Concentration (HN) | Concentration (LN) | Concentration (N removed) |
| --- | --- | --- | --- | --- |
| A | KNO_3_ | 2.5 mM | 0.25 mM | 0 mM |
| B | MgSO_4_ | 1 mM | 1 mM | 1 mM |
| C | Ca(NO_3_)_2_ | 1 mM | 0.1 mM | 0 mM |
| D | NH_4_NO_3_ | 1 mM | 0.1 mM | 0 mM |
| E | KH_2_PO_4_ | 1 mM | 1 mM | 1 mM |
| F | Fe-EDTA | 25 μM | 25 μM | 25 μM |
| G | H_3_BO_3_ | 35 μM | 35 μM | 35 μM |
|  | MnCl_2_ | 7 μM | 7 μM | 7 μM |
|  | CuSO_4_ | 0.25 μM | 0.25 μM | 0.25 μM |
|  | ZnSO_4_ | 0.5 μM | 0.5 μM | 0.5 μM |
|  | Na_2_MoO_4_ | 0.1 μM | 0.1 μM | 0.1 μM |
| H | NaCl | 5 μM | 5 μM | 5 μM |
| I | KCl | 0 mM | 2.25 mM | 2.5 mM |
| J | CaCl_2_ | 0 mM | 0.9 mM | 1 mM |

**Table S2**

Nitrogen concentrations, nitrogen masses, and dry weights of camelina plant organs in the HN-continued treatment at plant maturity (14 weeks after the onset of flowering). Mean values (n = 10) and error bars representing a 95% confidence interval are shown.

|  | Nitrogen (%) | Nitrogen (g plant^-1^) | Dry Weight (g plant^-1^) |
| --- | --- | --- | --- |
| **Shoot System** | 0.87 ± 0.18 | 0.22 ± 0.04 | 25.32 ± 2.07 |
| **Silicle** | 1.22 ± 0.13 | 0.08 ± 0.02 | 6.91 ± 1.11 |
| **Seed** | 4.46 ± 0.16 | 0.43 ± 0.07 | 9.59 ± 1.41 |

**Table S3**

Comparisons of residual leaf, shoot, and silicle nitrogen concentrations and NHI. Values for camelina are from variety ‘Suneson’ in this study; values for canola and Arabidopsis were obtained from the studies listed in footnotes. All values are reported at maturity except residual leaf nitrogen in HN-continued camelina, which is reported at week six post-flowering.

|  | **Residual Leaf Nitrogen** | **Residual Shoot Nitrogen** | **Residual Silicle/Pod Nitrogen** | **NHI** |
| --- | --- | --- | --- | --- |
| **Camelina; HN-continued** | 1.0%-1.7% | 0.87% | 1.22% | 0.59 |
| **Camelina; HN-removed** | 0.60%-0.74% | 0.43% | 0.41% | 0.68 |
| **Camelina; LN-continued** | 0.66%-0.95% | 1.18% | 0.71% | 0.61 |
| **Camelina; LN-removed** | 0.60%-0.95% | 0.27% | 0.41% | 0.72 |
| **Canola; high nitrogen** | 2.0%-4.5%^1,2,3,4^ | > 1%^2,6^ | ~ 2%^2,4,6^ | < 0.4^8,9^ |
| **Canola; low nitrogen** | ~ 1%^1,2,4^ | < 1%^2^ | < 1%^2,4^ | < 0.6^8,9^ |
| **Arabidopsis; high nitrogen** | ~ 4.5%^5^ | ~ 3.5%^5,7^ | no data | ~ 0.25^7,10^ |
| **Arabidopsis; low nitrogen** | ~ 0.7%^5^ | > 1%^5,7^ | no data | ~ 0.5^7,10^ |

^1.^ Dejoux JF, Recous S, Meynard JM, Trinsoutrot I, Leterme P. The fate of nitrogen from winter-frozen rapeseed leaves: mineralization, fluxes to the environment and uptake by rapeseed crop in spring. Plant Soil. 2000 Jan 1;218(1):257–72.

^2.^ Gombert J, Le Dily F, Lothier J, Etienne P, Rossato L, Allirand JM, et al. Effect of nitrogen fertilization on nitrogen dynamics in oilseed rape using ^15^N-labeling field experiment. J Plant Nutr Soil Sci. 2010;173(6):875–84.

^3.^ Rossato L, Lainé P, Ourry A. Nitrogen storage and remobilization in Brassica napus L. during the growth cycle: nitrogen fluxes within the plant and changes in soluble protein patterns. J Exp Bot. 2001 Aug 1;52(361):1655–63.

^4.^ Gammelvind LH, Schjoerring JK, Mogensen VO, Jensen CR, Bock JGH. Photosynthesis in leaves and siliques of winter oilseed rape (Brassica napus L.). Plant Soil. 1996 Oct 1;186(2):227–36.

^5.^ Chen Q, Soulay F, Saudemont B, Elmayan T, Marmagne A, Masclaux-Daubresse C. Overexpression of ATG8 in Arabidopsis stimulates autophagic activity and increases nitrogen remobilization efficiency and grain filling. Plant Cell Physiol. 2019 Feb 1;60(2):343–52.

^6.^ Bieker S, Riester L, Doll J, Franzaring J, Fangmeier A, Zentgraf U. Nitrogen supply drives senescence-related seed storage protein expression in rapeseed leaves. Genes. 2019 Jan 22;10(2):72.

^7.^ Guiboileau A, Yoshimoto K, Soulay F, Bataillé MP, Avice JC, Masclaux-Daubresse C. Autophagy machinery controls nitrogen remobilization at the whole-plant level under both limiting and ample nitrate conditions in Arabidopsis. New Phytol. 2012;194(3):732–40.

^8.^ Akmouche Y, Cheneby J, Lamboeuf M, Elie N, Laperche A, Bertheloot J, et al. Do nitrogen- and sulphur-remobilization-related parameters measured at the onset of the reproductive stage provide early indicators to adjust N and S fertilization in oilseed rape (Brassica napus L.) grown under N- and/or S-limiting supplies? Planta. 2019 Dec;250(6):2047–62.

^9.^ He H, Xie Y, Zhao A, Hu W, Guo X, Miller AJ, et al. Genotypic variation in nitrogen utilization efficiency in oilseed rape is related to the coordination of leaf senescence and root N uptake during reproductive stage. Plant Soil. 2021 June;463(1–2):291–306.

^10.^ Masclaux-Daubresse C, Chardon F. Exploring nitrogen remobilization for seed filling using natural variation in Arabidopsis thaliana. J Exp Bot. 2011 Mar 1;62(6):2131–42.


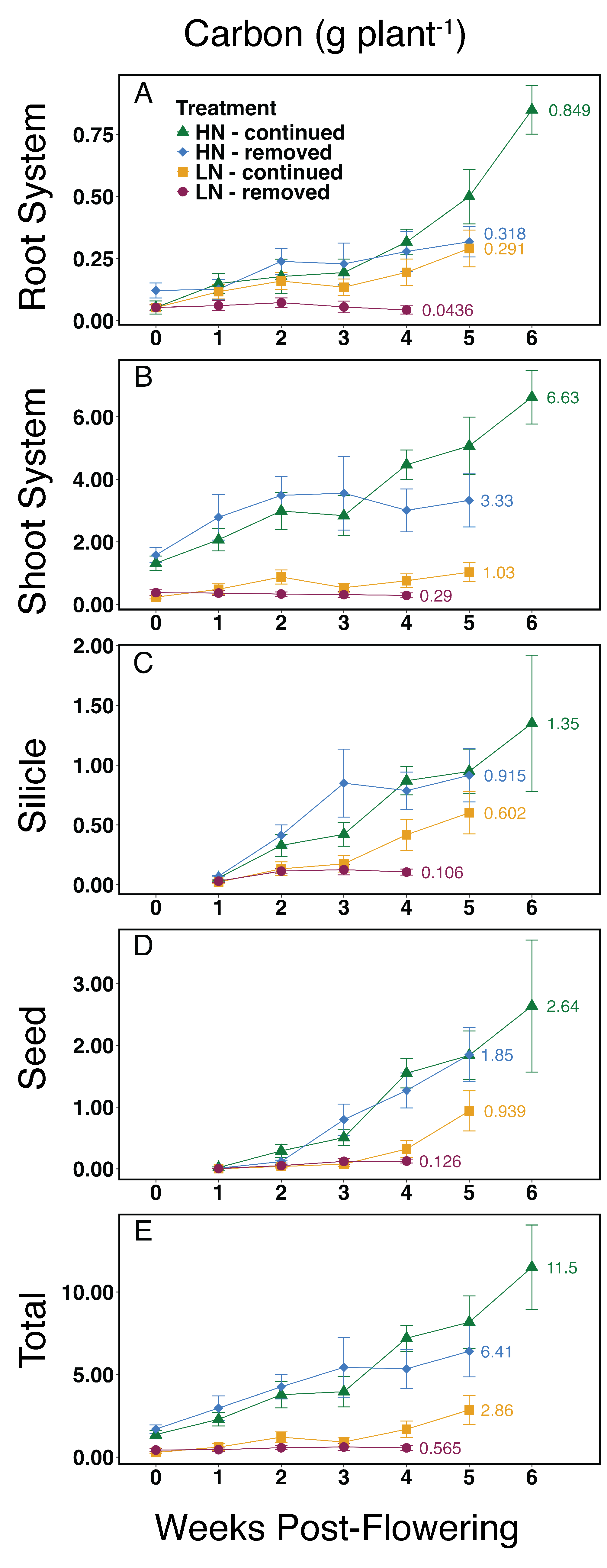


**Fig. S1**

Carbon masses in camelina plant organs are shown in weekly intervals, starting with the onset of flowering (week 0). A, roots systems; B, shoot systems including all stems and leaves; C, silicles (fruit pericarp); D, seeds; E, total plant carbon mass (sums of A-D). Mean values (n = 10) and error bars representing a 95% confidence interval are shown.


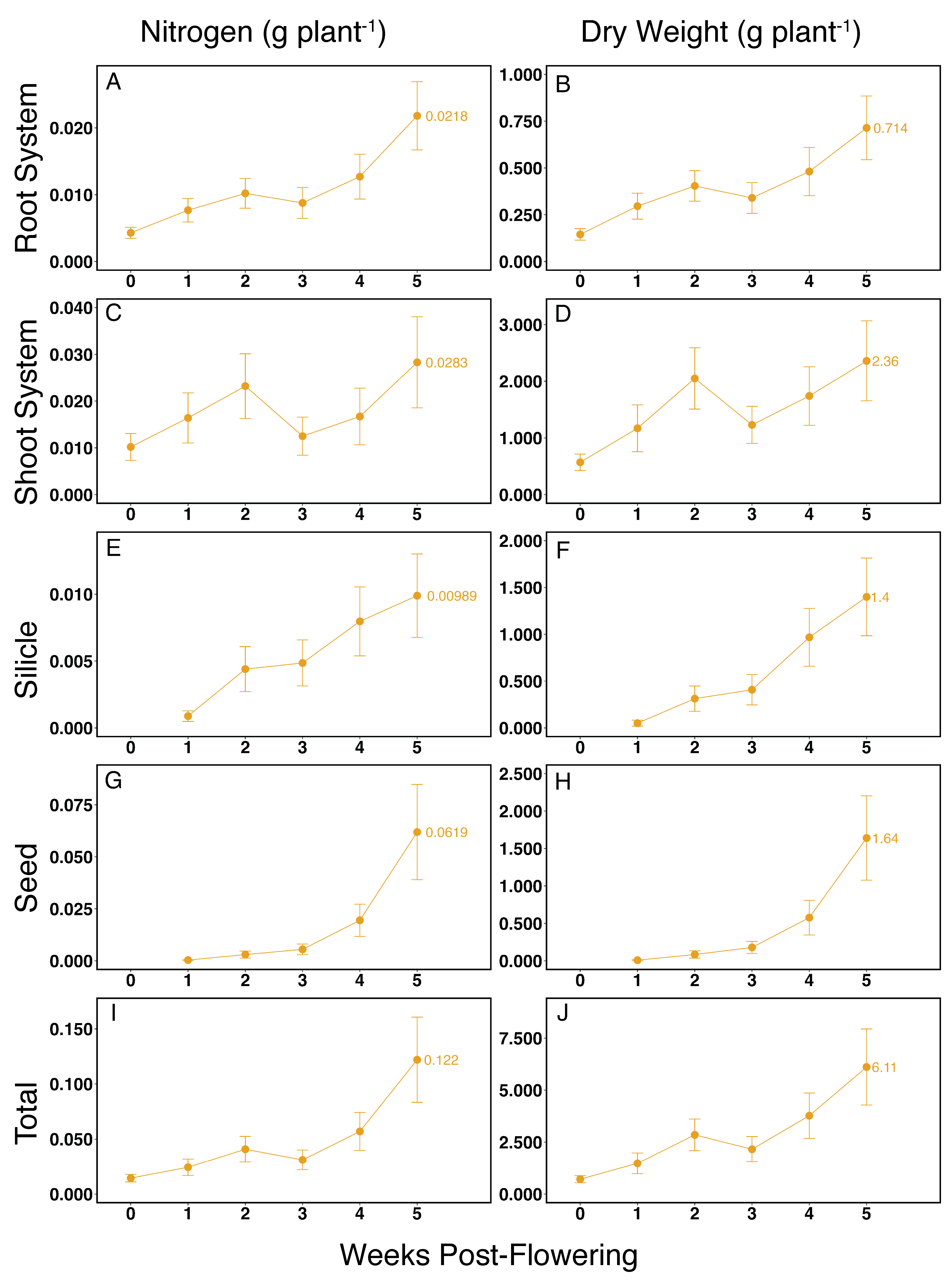


**Fig. S2**

Nitrogen mass and dry weight (biomass) in camelina plant organs are shown in weekly intervals for the LN-continued treatment, starting with the onset of flowering (week 0). Left panels show nitrogen mass, and right panels show dry weight. A, B, root systems; C, D, shoot systems including all stems and leaves; E, F, silicles (fruit pericarp); G, H, seeds; I, J, total plant nitrogen mass and dry weight. Mean values (n = 10) and error bars representing a 95% confidence interval are shown.


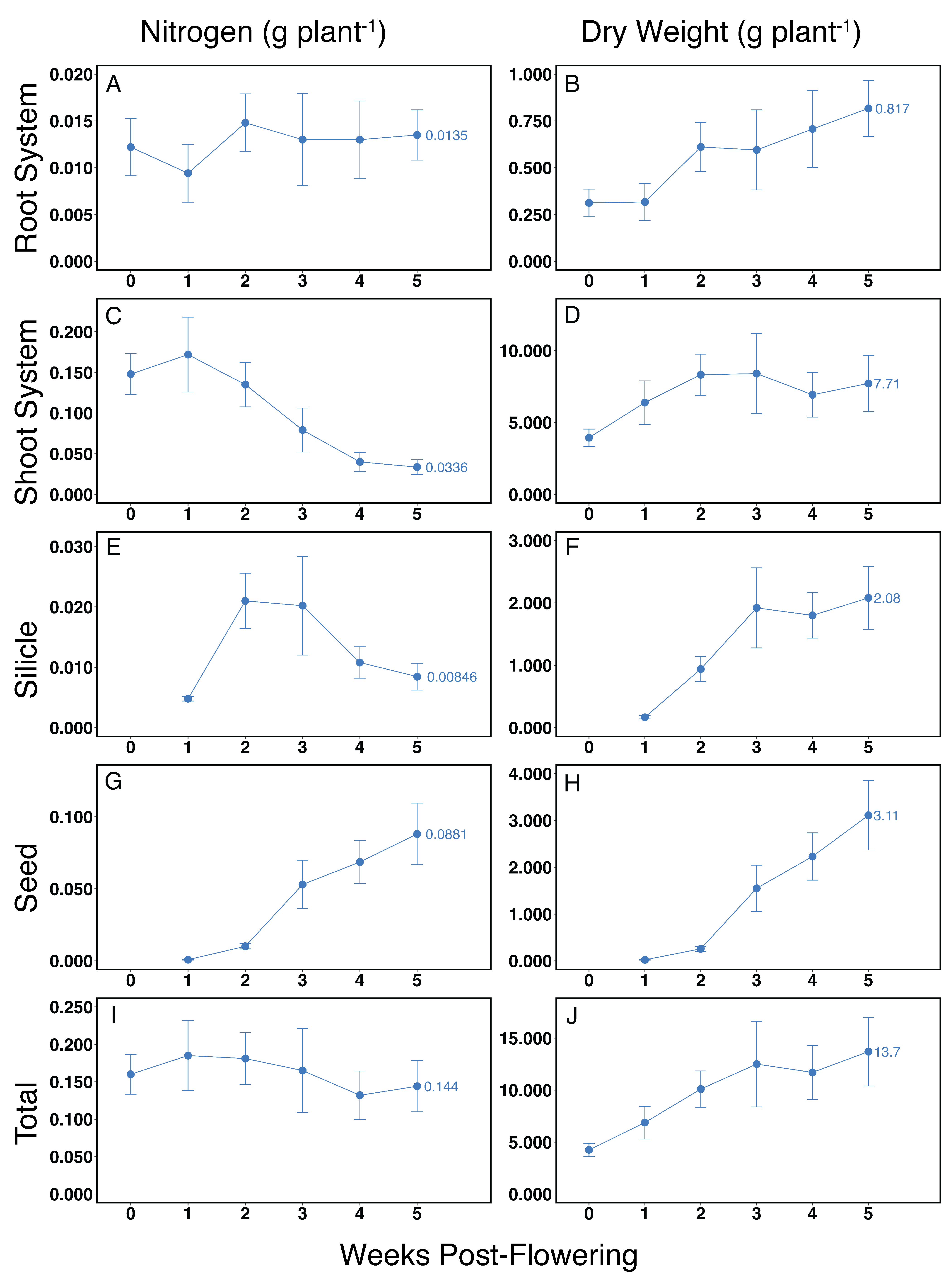


**Fig. S3**

Nitrogen mass and dry weight (biomass) in camelina plant organs are shown in weekly intervals for the HN-removed treatment, starting with the onset of flowering (week 0). Left panels show nitrogen mass, and right panels show dry weight. A, B, root systems; C, D, shoot systems including all stems and leaves; E, F, silicles (fruit pericarp); G, H, seeds; I, J, total plant nitrogen mass and dry weight. Mean values (n = 10) and error bars representing a 95% confidence interval are shown.


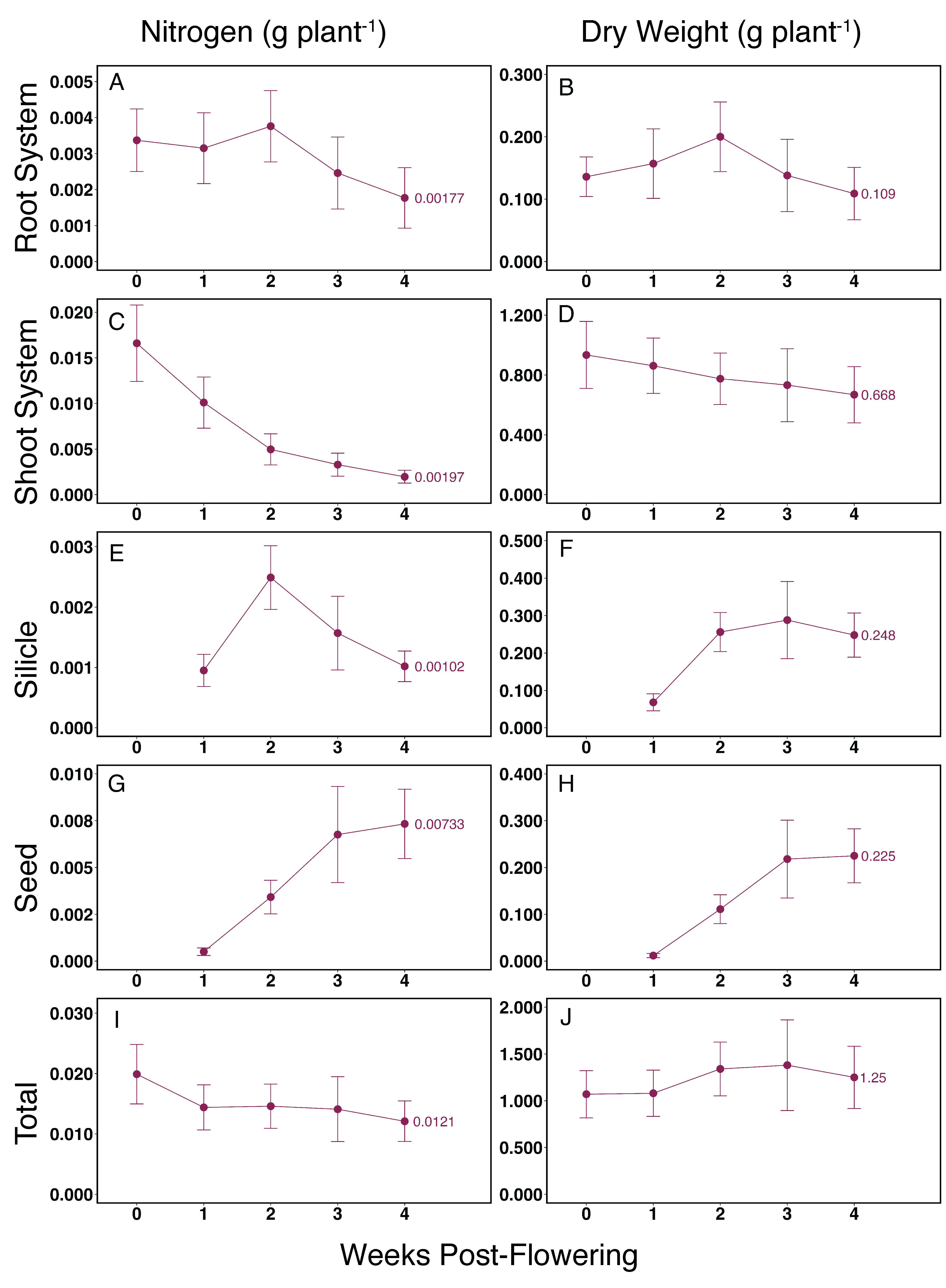


**Fig. S4**

Nitrogen mass and dry weight (biomass) in camelina plant organs are shown in weekly intervals for the LN-removed treatment, starting with the onset of flowering (week 0). Left panels show nitrogen mass, and right panels show dry weight. A, B, root systems; C, D, shoot systems including all stems and leaves; E, F, silicles (fruit pericarp); G, H, seeds; I, J, total plant nitrogen mass and dry weight. Mean values (n = 10) and error bars representing a 95% confidence interval are shown.


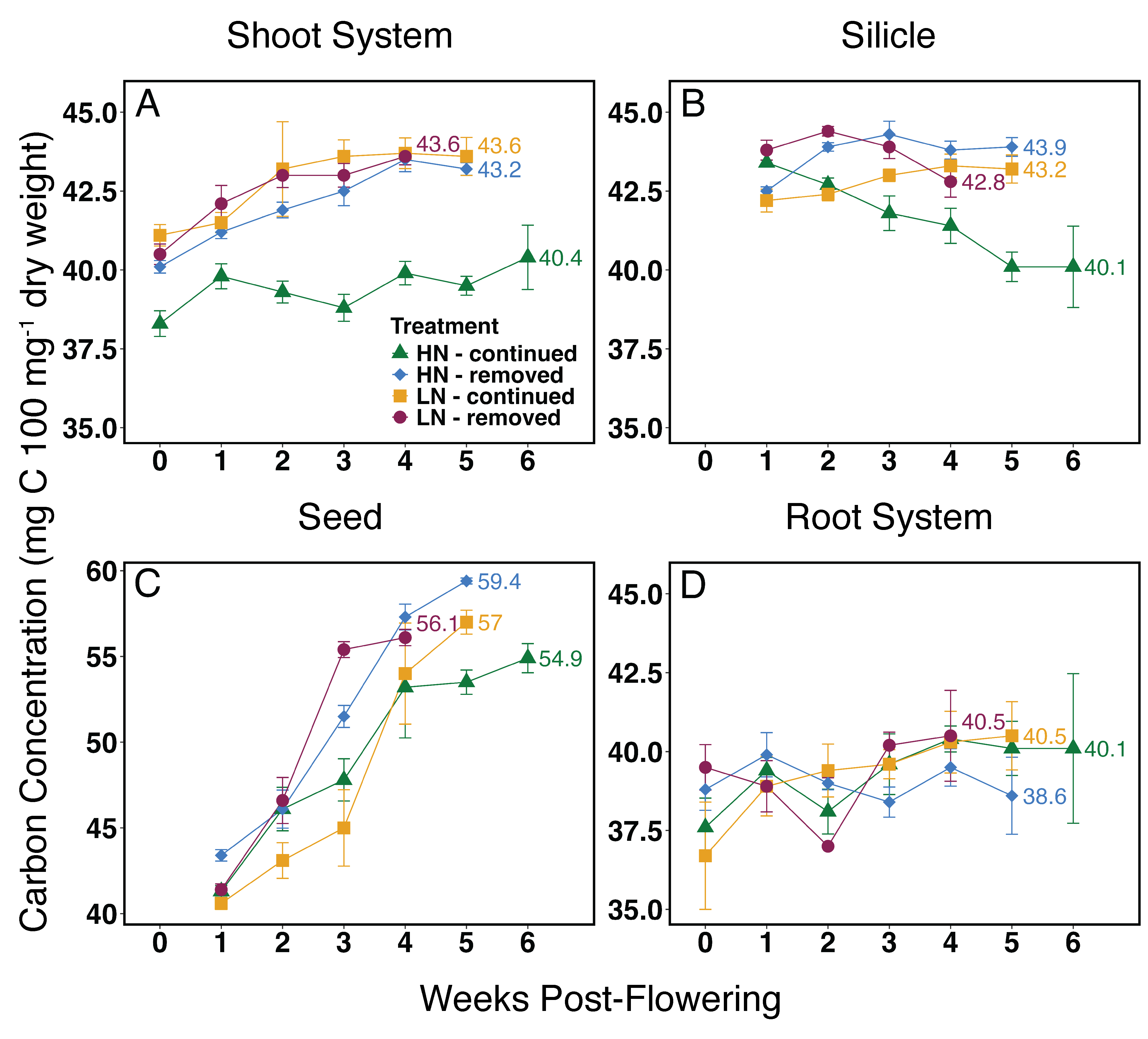


**Fig. S5**

Carbon concentrations in camelina plant organs are shown in weekly intervals, starting with the onset of flowering (week 0). A, shoot systems including all stems and leaves; B, silicles (fruit pericarp); C, seeds; D, root system. Mean values (n = 10) and error bars representing a 95% confidence interval are shown.
